# The impact of physical activity on substance use experimentation and initiation among adolescents: Results from the ABCD Study® cohort

**DOI:** 10.1016/j.dadr.2025.100373

**Published:** 2025-08-18

**Authors:** Christine M. Kaiver, Erin L. Thompson, Samuel W. Hawes, Sarah M. Lehman, Ashley R. Adams, David Wing, Angela R. Laird, Raul Gonzalez

**Affiliations:** a Center for Children and Families, Florida International University, Academic Health Center 1, 11200 SW 8th St Room 140, Miami, FL 33199, United States; b Herbert Wertheim School of Public Health and Human Longevity Science, University of California, San Diego, 9500 Gilman Drive, La Jolla, CA 92093, United States; c Department of Physics, Florida International University, FL, 11200 SW 8th Street, Miami, FL 33199, United States

**Keywords:** Physical activity, Substance use, Substance use initiation, ABCD Study, Adolescence

## Abstract

Physical Activity (PA) is important for mental, physical, and brain health. Adolescence is marked by increased engagement in risky substance use (SU) behaviors, which can negatively affect brain development. This study aims to determine if PA influences SU experimentation and initiation among adolescents. We predicted higher levels of PA would be associated with less SU, with a larger effect in more vigorous compared to light PA. A sample of 2541 participants from the Adolescent Brain Cognitive Development (ABCD) Study provided three weeks of Fitbit-measured PA data at the 2-year follow-up, and SU outcomes at the 3- and 4-year follow-up. SU outcomes of experimentation (i.e., sip/puff/try of alcohol, nicotine, or cannabis) and initiation (i.e., full drink of alcohol, more than a puff/try of nicotine or cannabis, or anything else) were examined dichotomously (i.e., yes/no). Logistic regression analyses were conducted, controlling for demographics, externalizing, and depressive symptoms endorsed on Child Behavior Checklist (CBCL). Total PA was associated with 24 % decreased odds in SU initiation (OR 0.82, 95 % CI 0.69–0.99, *p* < .05). After examining PA intensities more closely, light PA predicted 26 % decreased odds of SU initiation (OR 0.73, 95 % CI 0.61–0.88, p = .001). No significant associations emerged between PA and experimentation, or moderate and vigorous PA and initiation. More engagement in total and light PA reduced the odds of SU initiation, suggesting that low-intensity activity, not moderate or vigorous PA, may provide protection against adolescent SU. Future research should examine underlying mechanisms and contextual factors that account for these results.

## Highlights

• Objective physical activity (PA) data is a predicter of early adolescent substance use outcomes.
• No association found between PA and initiation of early use.
• Light, but not vigorous, PA predicts lower odds of more substance use involvement.

## 1 Introduction

Physical activity (PA) is linked to better physical, mental, and cognitive health across the lifespan (Ruegsegger and Booth, 2018). More frequent PA is associated with better mental health (Chekroud et al., 2018), less depression, self-harm, suicide attempts (Grasdalsmoen et al., 2020), and anxiety (Mikkelsen et al., 2017) and PA also improved performance on working memory, cognitive flexibility, and inhibitory control (Alves et al., 2012, Xiong et al., 2021).

Adolescence is a period of significant brain and cognitive development (Dumontheil, 2016; Luna and Sweeny, 2004) and increased SU risk (Gardner and Steinberg, 2005, Johnston et al., 2023). Studies on PA and adolescent SU show mixed findings. Some report PA is associated with less nicotine and cannabis use, but more alcohol use (McCaul et al., 2004, Terry-McElrath et al., 2011). Others have linked more PA with greater frequency of cannabis use (Pacheco-Colón et al., 2021). Current studies largely rely on self-reported PA, despite known discrepancies between self-report and objectively measured PA using accelerometry (Domingos et al., 2021, Katzmarzyk et al., 2017, Skender et al., 2016).

Several neurobiological mechanisms may underlie the protective effects of PA. Preclinical literature suggests PA enhances learning and memory by increasing Brain-Derived Neurotrophic Factor, long-term potentiation, and neurogenesis (Berchtold et al., 2005; Pereira, et al., 2007; Van Praag et al., 1999), which may reverse alcohol-related negative effects (Helfer et al., 2009, Maynard and Leasure, 2013). PA triggers the release of neurotransmitters and endogenous opioids, potentially creating a natural reward, particularly with vigorous PA (Nock et al., 2017), that could offer an alternative natural reward buffering against substance use (SU) initiation. Compared to light and moderate PA, one study found adolescents who engaged in vigorous PA reported less marijuana use (Delisle et al., 2010). However, further research is needed, especially during early adolescence, a key window for preventing substance use disorders (SUD) (Giesen, Deimel, and Bloch, 2015; Zschucke et al., 2012).

This study examined how objectively measured PA among early adolescents (via accelerometry and heart rate) is associated with two key stages of early SU: experimentation and subsequent initiation or escalation of use, using a subsample (n = 2541) from the Adolescent Brain Cognitive Development℠ Study (ABCD Study®). We tested whether total PA and specific PA intensities predicted SU outcomes. We hypothesized that more total PA would predict lower odds of future SU experimentation and initiation. Given the benefits of moderate- and vigorous-intensity PA (MVPA) (Nakagawa et al., 2020), we expected vigorous PA to have a stronger protective effect on SU outcomes than light PA.

## 2 Methods

### 2.1 Study procedure and participants

Data is from the ABCD Study® (Data Release 5.1; DOI: 10.15154/z563-zd24) from the National Institute of Mental Health Data Archive. Participants ages 9–10 were recruited across 21 sites in the U.S. between 2016 to 2018. Participants completed annual in-person visits and biannual phone interviews (details in Garavan et al., 2018). We used the first wave of PA data collected at the 2-year follow-up, and SU outcomes at the 3- and 4-year follow-ups. Participants were, on average, 9.9 years-old (SD =0.63) at baseline, 10.9 years-old (SD=0.64) at Year 1, 11.9 years-old (SD=0.65) at Year 2, 12.9 years-old (SD=0.65) at Year 3, and 14.0 years-old (SD=0.68) at Year 4. Study procedures were approved by the centralized institutional review board (IRB) at the University of California, San Diego and each local site’s IRB.

### 2.2 Measures

*Physical activity*

PA was assessed using Fitbit Charge Series devices worn for 21 days (Bagot et al., 2018). Fitbit data were validated against gold standard devices in children and adolescents (Bagot et al., 2018). Following ABCD Study® criteria, valid days included ≥ 599 min of waking wear time and ≥ 1000 steps per day to reflect a full day of activity, and ≥ 4 valid days reflected a valid week of activity (Wing et al., 2022). PA intensity was quantified using metabolic equivalents (METs), based on CDC classifications (Wing et al., 2022), which estimate energy expenditure relative to resting metabolic rates, thus providing the absolute intensity of various PA (Ferguson, 2014, Welk et al., 2019). Activity was categorized as light (1.5–3.0 METs), moderate (3.0–6.0 METs), or vigorous (>6.0 METs) using standardized cutoffs (Welk et al., 2019). Weekly average minutes were calculated for each intensity, and total PA was computed by averaging the intensity-specific averages.

*Substance use*

Youth reported lifetime SU at the baseline assessment across major drug categories. At each subsequent visit, youth reported SU since the previous follow-up. Among early adolescents, the most reported substances were alcohol, tobacco, and cannabis. Initial experiences using these substances were assessed using the iSay Sipping Inventory, Tobacco Low-Level Use, and Marijuana Low-Level Use, and follow-up questions gathered additional information about further SU initiation (Lisdahl et al., 2018). The current study adopted the ABCD Study® cohort’s SU classification approach from Sullivan et al. (2022), defining: *experimentation*: alcohol sipping or puff/trying nicotine/cannabis and *initiation*: reported ≥ 1 standard alcohol drink, > 1 puff/taste nicotine/cannabis, or any other occasion of other SU (Sullivan et al., 2022). To capture early SU and account for low SU prevalence, all substance types were collapsed into a single binary variable indicating any use. That is, participants were assigned to one of three groups based on their highest level of use, and regardless of the number of substances used.

*Covariates*

Potential confounders were included as covariates in all models based on prior literature supporting their association with SU outcomes: age, sex, race/ethnicity, parental income, parental education, parental history of problematic alcohol and/or SU, and the Child Behavior Checklist (CBCL) externalizing and depression scales (Sullivan et al., 2022).

### 2.3 Data analysis

Participants were excluded who either did not participate in the Fitbit portion of the study (*n = 683*), reported SU before the 2-year follow-up (*n = 2742*), or were missing SU data (*n = 4512*). The missingness reflects both the partial release of 4-year data in ABCD Study® Data Release 5.1 and participants with entirely missing SU data. Participants who opted to participate in the Fitbit protocol were previously reported to differ demographically from those who did not (Kim et al., 2023). To examine objective PA as a prospective predictor of low-level SU initiation, participants who reported SU prior to the Fitbit data collection were excluded. Those excluded were significantly older, had more externalizing and depressive symptoms, more likely to be male, higher parental education, higher household income, and problematic parental drug use (all p values <.05; see Table 1). Analyses were conducted using R software 4.3.1 (https://www.R-project.org/). Logistic regression models were used to examine the association between PA at the 2-year-follow-up and subsequent SU experimentation or initiation at the 3- and 4-year-follow-ups (Pampel, 2000). SU was classified as experimenter or initiator = 1 and Substance-use-naïve participants = 0 (Sullivan et al., 2022). Four models were conducted: 1) total PA predicting SU experimentation, 2) light, moderate, and vigorous PA predicting SU experimentation, 3) total PA predicting SU initiation, and 4) light, moderate, and vigorous PA predicting SU initiation. To address the wide PA range, winsorization at the 95th percentile was used to reduce the influence of extreme values while preserving rank order. These were then z-scored to facilitate interpretation and model convergence.

**Table 1:** Sociodemographic factors of sample.

M (SD) [Range] or N (%)	Substance-use-naïve *N = 2092*	Experimenters *N = 284*	Initiators *N = 165*	Group Differences (F/ χ²(df), p)	Excluded for SU Prior to Fitbit Collection *N = 2742*	Included vs. Excluded t or χ²(df), p)
Substance Use Prevalence Rates						
Alcohol	-	215 (8.5 %)	59 (2.3 %)			
Nicotine	-	16 (0.6 %)	109 (4.3 %)			
Cannabis	-	11 (0.4 %)	60 (2.4 %)			
Other Illicit Drugs	-	-	15 (0.6 %)			
Age (years)	14.0(0.67)	13.7(0.87)	13.9(0.76)	*F*(2, 2539) = 24.95, *p < .001*	13.8(0.84)	*t*(1475) = −3.33, *p = .0009*
Baseline Predictors						
Sex				χ²(2) = 6.81, *p = .03*		χ²(1) = 0.04, *p = .84*
Female	1047 (50 %)	157 (55.3 %)	97 (58.8 %)		1176 (42.9 %)	
Male	1045 (50 %)	127 (44.7 %)	68 (41.2 %)		1564 (57 %)	
Race				χ²(8) = 13.53, *p = .09*		χ²(4) = 30.08, *p < .001*
White	1117 (56 %)	168 (59.2 %)	80 (48.5 %)		1676 (61.1 %)	
Black	219 (10.5 %)	20 (7 %)	19 (11.5 %)		240 (8.8 %)	
Asian	55 (2.6 %)	< 10 (< 2 %)	< 10 (< 2 %)		49 (<2 %)	
Other Race(s)	203 (9.7 %)	37 (13 %)	22 (13.3 %)		299 (10.9 %)	
Ethnicity						
Hispanic/ Latino/a	444 (21.2 %)	54 (19 %)	42 (25.5 %)		477 (17.4 %)	
Household Income				χ²(4) = 18.95, *p < .001*		χ²(2) = 26.54, *p < .001*
< $50k	531 (25.4 %)	56 (19.7 %)	57 (34.5 %)		583 (21.3 %)	
$50–100k	615 (29.4 %)	71 (25 %)	46 (27.9 %)		685 (25 %)	
>$100k	946 (45.2 %)	157 (55.3 %)	62 (37.6 %)		1474 (53.8 %)	
Parental Education				χ²(8) = 31.40, *p < .001*		χ²(4) = 21.02, *p = .0003*
< HS Diploma	78 (3.7 %)	< 10 (< 5 %)	< 10 (< 5 %)		65 (<5 %)	
HS Diploma/GED	208 (10 %)	16 (5.6 %)	26 (15.8 %)		264 (9.6 %)	
Some College	621 (29.7 %)	80 (28.2 %)	65 (39.4 %)		726 (26.4 %)	
Bachelor	634 (30.3 %)	90 (31.7 %)	29 (17.6 %)		826 (30.1 %)	
Post Graduate Degree	551 (26.3 %)	92 (32.4 %)	38 (23 %)		856 (31.2 %)	
Parental History of Alcohol Use Related Consequences	279 (13.3 %)	46 (16.3 %)	41 (24.7 %)	χ²(2) = 16.17, *p < .001*	426 (15.5 %)	χ²(1) = 3.88, *p = .05*
Parental History of Drug Use Related Consequences	194 (9.3 %)	24 (8.6 %)	37 (22.3 %)	χ²(2) = 28.01, *p < .001*	314 (11.5 %)	χ²(1) = 4.80, *p = .03*
Mental Health CBCL (t-score)						
Externalizing Symptoms	44.9 (9.85)	45.2 (9.29)	48.3 (10.8)	F(2, 2539) = 8.93, p = .001	46.6 (10.5)	*t*(3132.8) = 3.50, *p = .0004*
Depressive Symptoms	53.4 (5.55)	52.9 (5.09)	54.8 (6.97)	*F*(2, 2539) = 6.55, *p = .001*	53.8 (5.75)	*t*(3136) = 1.38, *p = .17*
Physical Activity (Average minutes per week)						
Total	421 (134) [0–2241]	411 (122) [30−742]	391 (121) [76−666]	*F*(2, 2539) = 4.56, *p = .01*	404 (126) [0–1373]	*t*(2219) = −4.00, *p < .001*
Light	1124 (356) [0–6028]	1090 (331) [82–2125]	1030 (316) [152–1826]	*F*(2, 2539) = 06.33, *p = .002*	1064 (334) [0–3609]	*t*(2217) = −4.32, *p < .001*
Moderate	100 (84) [0−628]	98 (82) [0−466]	98 (84) [0−386]	*F*(2, 2539) = 0.11, *p = .90*	103 (82.1) [0−556]	*t*(2216.1) = −1.09, *p = .28*
Vigorous	40 (55) [0−442]	44 (60) [0−360]	46 (61) [0−345]	*F*(2, 2539) = 1.34, *p = .26*	44 (55) [0−517]	*t*(2196) = −0.47, *p = .64*

Notes: M, Mean. SD, Standard Deviation. Age corresponds to the 3-year or 4-year follow-up timepoint at which participants were classified as an experimenter, initiator, or the most recent follow-up visit for substance-use-naïve participants. Participants were assigned to one of three groups based on their highest level of use. HS, High School. GED, General Educational Development degree. Parent history of drug and alcohol use related consequences reported by parents at baseline. CBCL, Child Behavior Checklist is a parent-report questionnaire to assess internalizing symptoms and externalizing symptoms. Physical activity measured by Fitbit device. Welch’s two-sample t-tests and chi-square tests were used to compare participants excluded from the sample for early SU and participants retained in the sample, as well as to compare demographic differences between substance-use-naïve participants, experimenters, and initiators

Initial attempts to fit linear mixed-effects models to account for site-level and family-level nesting (Heeringa and Berglund, 2020) unable to converge due to nesting complexities. As a result, when participants had siblings in the study, one sibling per family was randomly retained in the final sample, resulting in *n = 2541*. To account for potential site-related effects, site ID was included as a fixed effect in all models. Missing data (<4 %) on covariates (i.e., parental education and parental history of problematic alcohol and SU) were imputed using multiple imputation via the mice (multivariate imputation by chained equations) package in R (van Buuren, 2018). Twenty imputed datasets were generated. Pooled estimates were used for final analyses.

## 3 Results

Table 1 presents descriptive statistics of the study sample. Correlations were examined to assess multicollinearity among predictor variables. Light, moderate, and vigorous PA were low to moderately correlated (*r* = .03–.61).

We first examined whether PA predicted increased odds of SU experimentation, adjusting for covariates (Table 2). Total PA did not significantly predict the odds of SU experimentation (*p = .96*). Light, moderate, and vigorous PA also showed no significant associations with experimentation (light: *p = .67*, moderate: *p = .80*, vigorous: *p = .18*). However, several covariates were significant. Older age (OR 1.79, 95 % CI 1.43–2.23, *p* < .001), more externalizing symptoms (OR 1.02, 95 % CI 1.01–1.04, *p = .002*), and female sex (OR 1.32, 95 % CI 1.01–1.73, *p = .04*) were associated with greater odds of SU experimentation, while depressive symptoms were negatively associated (OR 0.97, 95 % CI 0.94–1.00, *p = .03*).

**Table 2:** PA predicts SU experimentation and initiation.

	Model 1 Experimentation	Model 2 Experimentation	Model 3 Initiation	Model 4 Initiation
	OR [95 % CI]	OR [95 % CI]	OR [95 % CI]	OR [95 % CI]
Total PA	1.00 [0.87, 1.15]	-	0.82 [0.69, 0.99]*	-
Light PA	-	0.97 [0.84, 1.12]	-	0.73 [0.61, 0.89]**
Moderate PA	-	0.97 [0.79, 1.19]	-	1.16 [0.89. 1.51]
Vigorous PA	-	1.13 [0.95, 1.35]	-	1.12 [0.89, 1.41]
Significant Covariates				
Age	1.79 [1.43, 2.23]***	1.75 [1.39, 2.19]***	2.25 [1.67, 3.02]***	2.20 [1.62, 2.97]***
Sex (1 =male;2 =female)	1.32 [1.01, 1.73]*	1.42 [1.05, 1.93]*	1.49 [1.05, 2.12]*	1.96 [1.32, 2.92]***
Externalizing Symptoms	1.02 [1.01, 1.04]**	1.03 [1.01, 1.04]**	1.04 [1.01, 1.06]**	1.04 [1.01, 1.06]***
Depressive Symptoms	0.97 [0.94, 1.00]*	0.97 [0.94, 1.00]*	1.01 [0.98, 1.04]	1.01 [0.98, 1.04]
Parental History of Drug Use Related Consequences	0.82 [0.47, 1.44]	0.81 [0.46, 1.42]	1.80 [1.06, 3.05]*	1.81 [1.06, 3.08]*

*Notes:* OR =  odds ratio; CI =  confidence interval; p < .05 (*), p < .01 (**), p < .001(***). Only significant covariates are displayed. All models were adjusted for age, sex, race, ethnicity, externalizing symptoms, depressive symptoms, parent income, parent education, parental history of drug use related consequences, parental history of alcohol use related consequences, and site.

We next examined whether PA predicted increased odds of SU initiation, adjusting for covariates. Total PA was associated with 24 % lower odds of initiation (OR 0.82, 95 % CI 0.69–0.99, *p = .03*). Among PA intensities, light PA significantly predicted a 26 % decrease in odds of initiation (OR 0.73, 95 % CI 0.61–0.88, p = .001). Moderate (*p = .28)* and vigorous (*p = .32)* PA did not. Covariates significantly associated with initiation included higher age (OR 2.25, 95 % CI 1.67–3.02, p < .001), more externalizing symptoms (OR 1.04, 95 % CI 1.01–1.06, p = .001), female sex (OR 1.49, 95 % CI 1.05–2.12, *p = .02*), and parental history of drug use related consequences (OR 1.80, 95 % CI 1.06–3.05, *p = .03*).

## 4 Discussion

To our knowledge, this study is the first to examine whether objectively measured PA at varying intensities prospectively predicts early adolescent SU in the ABCD Study® cohort. Although the threshold for categorizing SU initiation was intentionally low to capture early use, the study identified significant associations between distinct PA intensities and SU. Total and light PA were significantly associated with reduced odds of SU initiation, suggesting that more frequent low-intensity activity may provide some protection against SU during adolescence. Contrary to expectations, PA (total, light, moderate, or vigorous) was not significantly associated with SU experimentation, reflecting PA may be more relevant to initiation than experimentation. It is also possible that several significant covariates may overshadow its effects. Increased age increased odds of both SU experimentation and initiation. As adolescents age, the protective factor of parental monitoring often decreases (Chassin et al., 2004; McLaughlin et al., 2016). Greater externalizing symptoms increased odds of SU experimentation and initiation, consistent with impulsivity and behavioral dysregulation as pathways to early SU (Miettunen et al., 2014). Parental history of drug use related consequences significantly predicted 80 % increased odds of SU initiation, highlighting intergenerational familial SU risk (Chassin et al., 1993).

Interestingly, more depressive symptoms were associated with lower odds of experimentation, but that finding diminished with SU initiation. This contrasts with existing literature linking depression to increased SU risk among older adolescents (Hussong et al., 2011). However, depressive symptoms are heterogeneous in nature (Chinet et al., 2006), and one possibility is that more withdrawn youth are less likely to engage in peer-driven behaviors like SU experimentation in early adolescence. Longitudinal studies need to explore how this relationship evolves among older adolescents. Surprisingly, females were more likely to engage in SU experimentation (32 % increased odds) and initiation (49 % increased odds) compared to males in this cohort. While this contrasts with historical SU trends, this aligns with recent research suggesting a narrowing gender gap in adolescent SU (Bhatia et al., 2023, Heitzeg et al., 2018). This may partly reflect greater peer involvement among girls, increasing their exposure to SU opportunities within social groups (Simons-Morton et al., 2001).

Mixed findings in the literature may stem from use of self-report measures. Many prior studies focused on self-reported PA and emphasize MVPA (Fagan et al., 2023; Pate et al., 2007), potentially overlooking the protective role of light PA. Some studies found associations between light PA and increased cannabis use and binge drinking (McCaul et al., 2004); others found more MVPA to be associated with lower odds of smoking compared to light PA (Davis et al., 1997). These inconsistencies highlight the need for future research to examine the mechanisms by which objectively measured PA intensity either buffers against or contributes to risk of SU in the context in which PA occurs (e.g., individual vs team sports) and if it differs by specific substance classes.

Several limitations should be considered. First, SU outcomes did not differentiate between substance types, use of multiple substances, or sports involvement alongside PA intensities, which could influence SU risk through peer interactions or pain-related motives. Second, participants retained in the sample significantly differed from those excluded. These differences may limit the generalizability to very early substance use. Although we used a prospective design, longitudinal research tracking this cohort into later age is needed to clarify how PA patterns influence SU trajectories across adolescence.

Objective PA metrics provide valuable data to capture both PA volume and intensity beyond that afforded by self-report. Given the influence PA has on cognitive functioning and reward-related pathways, future research should test whether PA influences SU risk via these pathways. Large-scale longitudinal studies are needed to test whether sustained PA engagement can delay or prevent the onset of problematic SU. This work may be essential to understanding whether PA can be leveraged to reduce the risk of SU in adolescence.

## CRediT authorship contribution statement

**Erin L. Thompson:** Writing – review & editing, Project administration, Formal analysis, Data curation. **M KAIVER CHRISTINE Marie:** Writing – original draft, Visualization, Project administration, Methodology, Investigation, Formal analysis, Data curation, Conceptualization. **Sarah M. Lehman:** Writing – review & editing, Project administration, Investigation. **Samuel W. Hawes:** Writing – review & editing, Project administration, Data curation. **David Wing:** Writing – review & editing, Methodology, Data curation. **Ashley R. Adams:** Writing – review & editing, Project administration, Investigation. **Angela R. Laird:** Writing – review & editing, Resources, Project administration, Funding acquisition. **Raul Gonzalez:** Writing – review & editing, Supervision, Resources, Project administration, Methodology, Funding acquisition, Conceptualization.

## Ethical standards

All participants provided informed consent or assent. The study was conducted complying with the ethical standards of the relevant national and institutional committees on human experiments and with the Declaration of Helsinki of 1975.

## Funding

The author(s) declare that financial support was received for the research and/or publication of this article. This research was supported by the National Institute of Health (10.13039/100000002NIH) awards U01DA041156 (PI: Gonzalez, R. and Laird, A.R.). Thompson, E.L. was supported by the National Institute on Minority Health and Health Disparities award K01MDO18069. Wing, D. was supported by the NIH award U24DA041147 (PI: Brown, S.A. and Jernigan, T.). Adams, A.R. was supported by National Institute of Drug Abuse award T32DA043449 (PI: Gonzalez, R.). Data used in the preparation of this article were obtained from the Adolescent Brain Cognitive Development (ABCD) Study® (https://abcd study.org), held in the NIMH Data Archive (10.13039/100009477NDA). This is a multisite, longitudinal study designed to recruit more than 10,000 children age 9–10 and follow them over 10 years into early adulthood. The ABCD Study® is supported by the National Institutes of Health and additional federal partners under award numbers U01DA041048, U01DA050989, U01DA051016, U01DA041022, U01DA051018, U01DA051037, U01DA050987, U01DA041174, U01DA041106, U01DA041117, U01DA041028, U01DA041134, U01DA050988, U01DA051039, U01DA041156, U01DA041025, U01DA041120, U01DA051038, U01DA041148, U01DA041093, U01DA041089, U24DA041123, U24DA041147. A full list of supporters is available at https://abcdstudy. org/federal-partners.html. A listing of participating sites and a complete listing of the study investigators can be found at https://abcdstudy.org/consortium_members/. ABCD consortium investigators designed and implemented the study and/or provided data but did not necessarily participate in the analysis or writing of this report. This manuscript reflects the views of the authors and may not reflect the opinions or views of the NIH or ABCD consortium investigators. The ABCD data repository grows and changes over time. The ABCD data used in this report came from ABCD Data Release 5.1 (https://data-archive.nimh.nih.gov/abcd; DOI: 10.15154/z563-zd24, December 2024).

## Declaration of Competing Interest

The authors declare the following financial interests/personal relationships which may be considered as potential competing interests: Christine M. Kaiver reports financial support was provided by Florida International University. Erin L. Thompson reports financial support was provided by National Institute on Minority Health and Health Disparities. Samuel W. Hawes reports financial support was provided by Florida International University. Sarah M. Lehman reports financial support was provided by Florida International University. Ashley R. Adams reports financial support was provided by National Institute on Drug Abuse. David Wing reports financial support was provided by University of California San Diego. Angela R. Laird reports financial support was provided by Florida International University. Raul Gonzalez reports financial support was provided by Florida International University. If there are other authors, they declare that they have no known competing financial interests or personal relationships that could have appeared to influence the work reported in this paper.
